# Relationship between volume of submandibular salivary stones in vivo determined with Cone-Beam Computer Tomography and in vitro with micro-Computer Tomography

**DOI:** 10.4317/medoral.24605

**Published:** 2021-08-19

**Authors:** Saskia Kraaij, Henk S Brand, Erik H van der Meij, Jan GAM de Visscher

**Affiliations:** 1 Department of Oral and Maxillofacial Surgery / Oral Pathology Amsterdam UMC location VUmc and Academic Centre of Dentistry Amsterdam (ACTA), Amsterdam, the Netherlands; 2 Department of Oral Biochemistry, Academic Centre for Dentistry Amsterdam (ACTA), Amsterdam, the Netherlands; 3 Department of Oral and Maxillofacial Surgery, Medical Centre Leeuwarden, Leeuwarden, the Netherlands

## Abstract

**Background:**

Successful removal of salivary stones depends on exact pretreatment information of the location, the size and shape of the stones. This study aimed to compare the volume of submandibular sialoliths determined by preoperative Cone-Beam Computer Tomography (CBCT) scans with the volume of the removed stones on micro-Computer Tomography (micro-CT) scans.

**Material and Methods:**

In this study, using twenty-one submandibular sialoliths, the pretreatment volumes in-vivo measured on CBCT were compared to the volumes of removed stones determined by micro-CT scans. The volume measured on micro-CT scans served as the gold standard. Pre-operative CBCT’s and in-vitro micro-CT’s were converted into standard tessellation language models (STL-models) using an image segmentation software package. The CBCT and micro-CT images of the stones were subsequently metrologically assessed and compared to each other using reverse engineering software.

**Results:**

Volumes of submandibular sialoliths determined by CBCT’s correlated significantly with volumes measured on micro-CT’s (Spearman’s coefficient r = 0.916). The interquartile range (IQR) for the volume measured with micro-CT was 117.23. The median is 26.41. For the volume measured with CBCT the IQR was 141.3 and the median 36.61. The average volume on micro-CT is smaller than on CBCT.

**Conclusions:**

When using CBCT-scans for the detection of submandibular sialoliths one should realize that in-vivo those stones are actually a fraction smaller than assessed on the preoperative scan. This is important when cut-off values of sizes of stones are used in the pretreatment planning of stone removal.

** Key words:**Salivary stone, sialolith, CBCT, micro-CT, volume.

## Introduction

Salivary stones are mineralized structures most often located in the efferent ducts of the submandibular and parotid glands and less often in the salivary gland itself. This may cause, frequently mealtime related, obstruction resulting in stasis of saliva causing recurrent swelling and pain of the affected salivary gland. In some persistent cases a bacterial sialoadenitis occur (1). Distribution of sialolithiasis in a large series, showed that 80% were located in the submandibular duct system (53% proximal/hilar, 37% distal, 10% intraparenchymal) and 20% in the parotid duct system (83% Stenson’s duct, 17% intraparenchymal) (2). For successful treatment of sialolithiasis, exact pretreatment information on the size, volume and location of the salivary stone are important so an informed choice can be made with regard to the most suiTable treatment modality. Over 50% of salivary stones cannot clinically be reliable assessed by palpation and/or location (2). Depending on the degree of calcification, some salivary stones can be identified as a radiopaque structure during radiographic examination, despite the relatively high percentage of inorganic material. Various imaging techniques are used to detect the possible presence of salivary stones in patients with recurrent obstructive disease of the submandibular or parotid gland such as occlusal radiograph, panoramic radiograph, sialography, ultrasonography (US), spiral computed tomography (CT) and cone-beam CT (CBCT) and magnetic resonance sialography. CT and CBCT scans are nowadays the preferred radiographic examination techniques for detecting the possible presence of salivary stones with a reported high sensitivity and specificity (3), whereby CBCT is more routinely practice because of the smaller radiation dose (30-80µSv) and lower purchasing costs (4). Micro-CT is basically a miniaturized version of a CT device optimized for the micron imaging but cannot be used for diagnostic examination because of the small scanning range.

The aim of the present study was to compare the volume of salivary stones determined by preoperative CBCT scans with the volume of the removed stones on micro-CT scans in series of submandibular sialolithiasis.

## Material and Methods

In the period from February 2013 to June 2016, in a consecutive series of patients at the department of Oral and Maxillofacial Surgery of the Medical Centre Leeuwarden, the Netherlands, there were twenty-one patients with submandibular salivary stones who had undergone a pretreatment CBCT scan, in an upright sitting position. The CBCT images were performed on a Vatech Panoramic X-Ray System PaX-Zenith 3D radiographic imaging device (Vatech, Gyeongg-do, Korea). The scanning parameters were set at 105kV and 4,5mA. In all cases a large field of view was used. The basic magnification [1,338] of the device when using a large field of view is automatically corrected by the accompanying software making the values of size and shape on the scans correspond to reality.

Before micro-CT imaging, the obtained stones were precisely placed in a medical glove and fixed using polyether impression material Impregum™ Penta™ (Pentamix 3, 3M ESPE, Seefeld, Germany). The fixed salivary stones were scanned using a micro-CT scanner, µCT 40 Scanco Medical (Wangen-Brüttisellen, Switzerland). The calculated micro-CT volume served as a ‘gold standard’ since the accuracy of a micro-CT device is very high (5).

All measurements obtained from the CBCT and micro-CT images were calculated using OsiriX (Pixmeo SARL, Bernex, Switzerland) and converted into 3D standard tessellation language (STL) file format surface models. The STL models were subsequently imported into GOM Inspect reverse engineering software (GOM GmbH, Braunschweig, Germany) where the distortion was removed and the volume and surface of each stone was measured. In a last step, all CBCT and their corresponding micro-CT STL models were superimposed on each other using GOM software to assess volume differences between the CBCT and micro-CT images.

Statistical analysis was performed with IBM SPSS Statistics for Windows version 26.0 (IBM Inc, Armonk, NY), using Wilcoxon signed rank test and Spearman’s rank order coefficient. P-values of 0.05 or less were considered statistically significant.

The current study followed the principles of the Helsinki Declaration and was performed in accordance with the guidelines of the Medical Ethic Committee of the Amsterdam UMC location VUMC (protocol number 2012/127).

## Results

**Figure 1:**
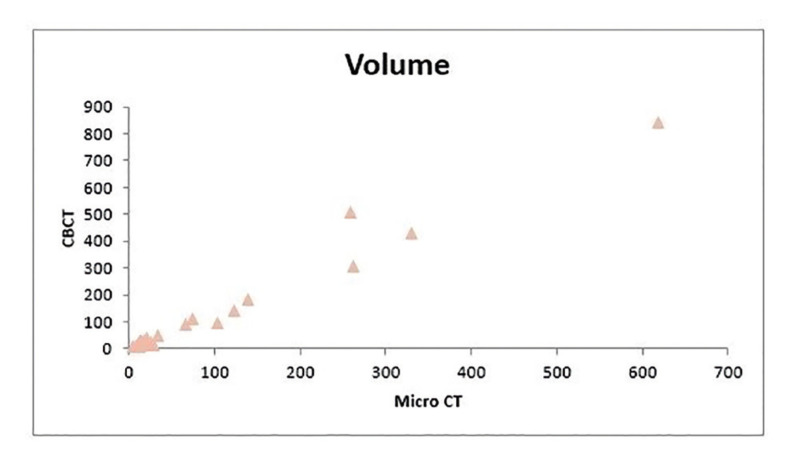
Relation between the volume determined by both CB-CT and micro-CT of 21 submandibular sialoliths. Data are expressed as mm3.

**Figure 2:**
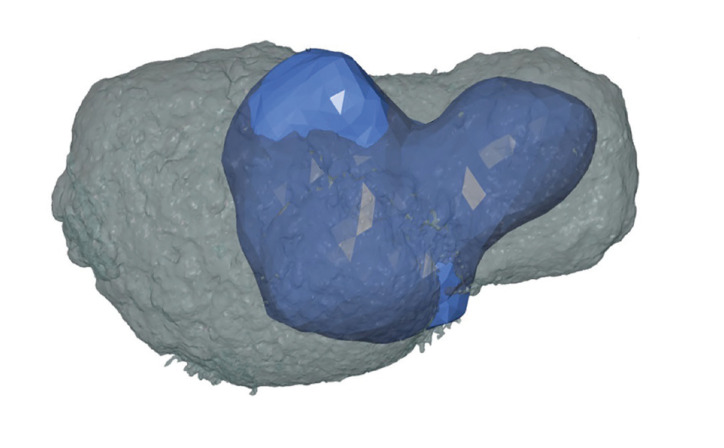
MUCT (blue, volume 258,94mm3) and CBCT (gray, volume 504,42mm3) STL-models projected on top of each other.

Sialoliths were derived from 14 females and 7 males with a mean age of 37 years (range 12-79). Fourteen sialolithiasis were located in the left and 7 in the right submandibular ductal system. The stones were removed by conventional surgery [7], sialendoscopy [10] and sialendoscopicaly assisted surgical approach (‘combined approach’) [4]. The characteristics of the study population are reported in Table 1. The mean volume of the 21 submandibular salivary stones on CBCT was 141,7 mm3 (range 8.1 - 840 mm3) with a median of 36.61 and an interquartile range of 141.3, which was significantly larger than the mean volume on micro-CT of 103,5 mm3 (range 4.5 - 619.1 mm3, median 26.41 and IQR 117.23). (Wilcoxon test *p* = 0.001). On average, submandibular stones measured 19.7% smaller on micro-CT than on the pre-operative CBCT. The volumes determined by CBCT correlated highly significant with the volumes determined with micro-CT (Spearman’s coefficient r = 0.916, *p* < 0.0005) (Fig. 1, Fig. 2).

**Table 1:**
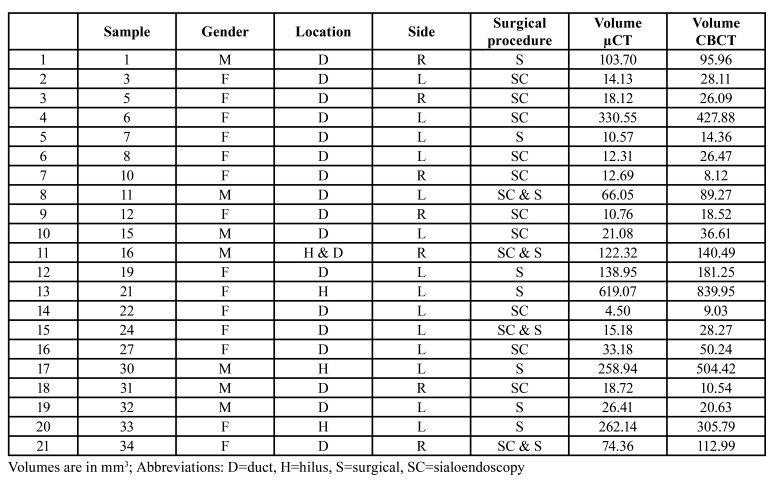
Characteristics of the study population.

## Discussion

Exact pretreatment information on the location, size, volume and shape of a salivary stone is essential to guide management and a wide variety of imaging modalities are available for this purpose e.g. MRI, extra oral OPT or conventional X-ray. Each of these imaging modalities has its own advantages and disadvantages with regard to the use of ionizing radiation, costs, availability, and capability to visualize the ductal system (1). The most-used techniques for the evaluation of the possible presence of salivary stones today are CBCT, US and conventional 2D radiography (3,6). Based on recent data, CBCT seems to be an imaging modality with high specificity and positive predictive value, and even higher sensitivity and negative predictive value. This high accuracy combined with low costs, high availability and limited radiation exposure makes CBCT an ideal first line imaging modality in patients with signs and symptoms of obstructed major salivary glands (3). US sensitivity for salivary stone detection is assumed to be around 75% (7). Failure has been reported in cases of small and semi-calcified stones. Calculi with a diameter of less than 3 mm are most often missed at US because they do not produce a dorsal acoustic shadow or because they are not hyperechoic with regard to surrounding structures. The lack of a dorsal acoustic shadow may depend not only on the size but also on the chemical composition of calculi. Besides, calculi within the distal duct are not shown accurately with US. Recently it was reported that ultrasound measurements of salivary stones in millimeters correlated highly with ex vivo measurements after removal (7,8). Conventional 2D radiography is still routinely used in daily practice nowadays. However, on panoramic radiographs, salivary stones can be missed because they may be projected superimposed on bony structures or teeth. In addition, occlusal and panoramic radiographs are two-dimensional imaging modalities, with concomitant limited possibilities to determine the volume and shape of the sialoliths (1,9).

Previous studies suggest that submandibular stones with a diameter of less than 4 mm may be manageable to sialendoscopical removal (10-12). Unfortunately, the practical value of the current used cut-off value is limited, due to the use of various imaging techniques and the fact that none of the studies indicated whether the cut-off diameter concerned the widest cross section or the longitudinal section.

The results of the present study suggest that when CBCT-scans are used for the detection of submandibular salivary stones one should realize that *in vivo* those stones are actually a fraction smaller than assessed on the preoperative CBCT-scan. This finding is particularly important when cut-off values of sizes of stones are used in the pretreatment planning of stone removal. A possible limitation of this study is the setting of the voxel size on the CBCT device. Volume measurements up to a voxel size of 200m (100m, 150m and 200) show no differences in measurements, despite a slight tendency towards underestimation, which increases with voxel size. At 300m and above, the underestimation of measurements becomes statistically significant (13,14). To overcome this limitation and to ensure that one measures the actual volume of the stone, it is recommended to use the smallest voxel size possible.

## References

[1] Kraaij S, Karagozoglu KH, Forouzanfar T, Veerman ECI, Brand HS (2014). Salivary stones: symptoms, aetiology, biochemical composition and treatment. Br Dent J.

[2] Sigismund PE, Zenk J, Koch M, Schapher M, Rudes M, Iro H (2015). Nearly 3,000 salivary stones; some clinical and epidemiologic aspects. Laryngoscope.

[3] van der Meij EH, Karagozoglu KH, de Visscher JGAM (2018). The value of cone beam computed tomography in the detection of salivary stones prior to sialendoscopy. Int J Oral Maxillofac Surg.

[4] Miracle AC, Mukherji SK (2009). Conebeam CT of the head and neck, Part 1: Physical principles. AJNR Am J Neuroradiol.

[5] Bouxsein ML, Boyd SK, Christiansen BA, Guldberg RE, Jepsen KJ, Müller JR (2010). Guidelines for assessment of bone microstructure in rodents using micro-computed tomography. J Bone Miner Res.

[6] Goncalves M, Schapher M, Iro H, Wuest W, Mantsopoulos K, Koch M (2017). Value of sonography in the diagnosis of sialolithiasis. Comparison with the reference standard direct stone identification. J Ultrasound Med.

[7] Terraz S, Poletti PA, Dulguerov P, Dfouni N, Becker CD, Marchal F (2013). How reliable is sonography in the assessment of sialolithiasis?. AJR Am J Roentgenol.

[8] Badger CD, Patel S, Romero NJ, Andrew Fuson A, Joshi AS (2021). In vivo accuracy of ultrasound for sizing salivary ductal calculi. Otolaryngol Head Neck Surg.

[9] Bodner L (2002). Giant salivary gland calculi: Diagnostic imaging and surgical management. Oral Surg Oral Med Oral Pathol Oral Radiol Endod.

[10] Marchal F, Dulguerov P (2003). Sialolithiasis management: the state of the art. Arch Otolaryngol Head Neck Surg.

[11] Walvekar RR, Carrau RL, Schaitkin B (2009). Endoscopic sialolith removal: orientation and shape as predictors of success. Am J Otolaryngol.

[12] Koch M, Zenk J, Iro H (2009). Algorithms for treatment of salivary gland obstructions. Otolaryngol Clin North Am.

[13] Yilmaz F, Sonmez G, Kamburoglu K, Koç C, Ocak M, Çelik HH (2019). Accuracy of CBCT images in the volumetric assessment of residual root canal filling material; Effect of voxel size. Niger J Clin Pract.

[14] Maret D, Telmon N, Peters OA, Lepage B, Treil J, Inglèse JM (2012). Effect of voxel size on the accuracy of 3D reconstructions with cone beam CT. Dentomaxillofacial Radiol.

